# Therapeutic strategies to overcome *ALK*-fusion and *BRAF*-mutation as acquired resistance mechanism in *EGFR*-mutated non-small cell lung cancer: two case reports

**DOI:** 10.3389/fonc.2024.1390523

**Published:** 2024-11-01

**Authors:** Yuan Zeng, Qiang Zeng, Bin Yang, Yang Hu

**Affiliations:** ^1^ Department of Oncology, Hubei Cancer Hospital, TongJi Medical College, Huazhong University of Science and Technology, Wuhan, China; ^2^ Department of Intensive Care Unit, Chengdu Shuangliu Hospital of Traditional Chinese Medicine, Chengdu, China

**Keywords:** EGFR-TKI resistance mechanisms, acquired ALK-fusion, acquired BRAF-mutation, NSCLC, case report

## Abstract

Non-small cell lung cancer (NSCLC) is one of the most common malignancies in the world. *EGFR* tyrosine inhibitors are the preferred first-line treatment for patients with epidermal growth factor-cell receptor mutant (*EGFR* mutant) advanced NSCLC. Unfortunately, drug resistance inevitably occurs leading to disease progression. Activation of the *ALK* and *BRAF* bypass signaling pathways is a rare cause of acquired drug resistance for *EGFR*-TKIs.We report two NSCLC-patients with *EGFR*- mutations, in exon 19, and exon 18, correspondingly, who were treated with *EGFR*-TKIs. The first case shows acquired *BRAF*-mutation, and the second case demonstrates acquired *ALK*-fusion. The overall survival of patients was significantly prolonged by drug-match therapies. As it is well-known that *ALK*-fusion and *BRAF*-mutations are described forms of acquired resistance. These two case reports contribute to the previous reports that *ALK*-fusion and *BRAF*-mutation are potential underlying mechanisms of *EGFR*-TKI resistance.

## Introduction

Non-small-cell lung cancer (NSCLC) is the most common type of lung cancer, accounting for approximately 80% -85% of the cases (1). In recent years, significant progress has been made in the molecular genetics of lung cancer, and the treatment of NSCLC has entered the era of targeted therapy (2). The most common driver gene mutation in NSCLC is the epidermal growth factor receptor (*EGFR*), which is found in 45% of Asian patients with adenocarcinoma histology and in 20% of Caucasian patients (3). The most common gene mutations include *EGFR* 19 del and *EGFR* 21 L858R. In patients with advanced NSCLC harboring sensitizing *EGFR* mutations, *EGFR*-tyrosine kinase inhibitor (*EGFR*-TKI) treatment was significantly better effective than conventional chemotherapy. Globally, neither treatment guidelines nor clinical practice considers *EGFR*-TKIs as the first-line treatment option for *EGFR*-mutated metastatic NSCLC patients (4).

However, the vast majority of patients inevitably develop acquired drug resistance, which seriously affects the survival prognosis of patients (5). The resistance mechanisms can be generally classified into two categories: on-target and off-target resistance. We report two cases of rare resistance mechanisms involving *ALK* and *BRAF* pathway that occurred under *EGFR*-TKI treatment. To some extent, our cases contribute to previously reported new treatment approaches.

## Case 1

**Figure 1:**
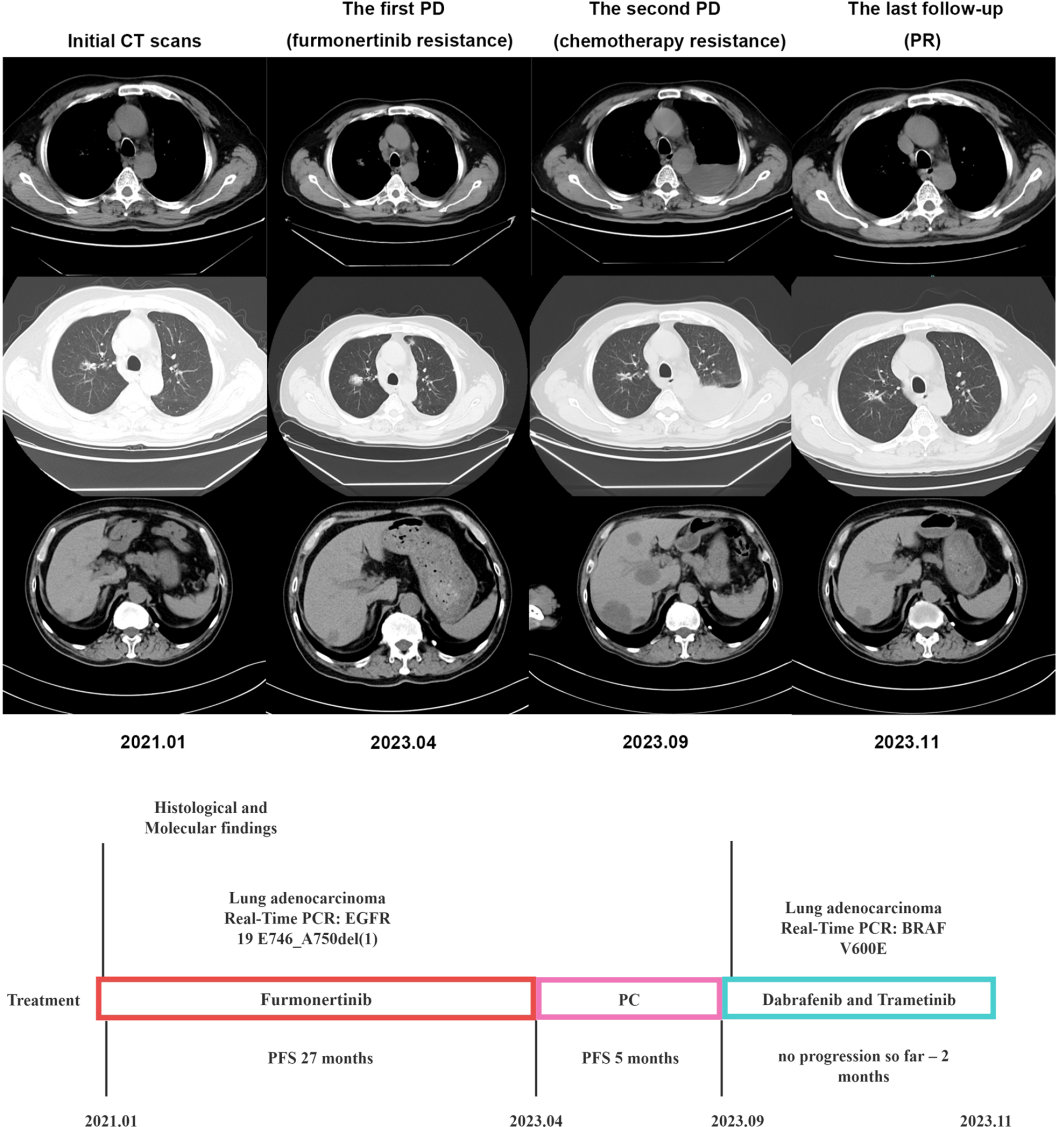
Timeline illustrating the changes in therapeutic regimen in correlation with molecular and radiological findings. PCR, digital polymerase chain reaction; PFS, progress free survival; PC, pemetrexed combined with cisplatin; PD, progression of disease; PR, partial remission.

A 60-year-old male smoker presented with complaints of cough and fatigue. A chest CT scan revealed multiple nodules in both lungs, with the largest measuring approximately 1.1x0.8cm, suggesting the presence of lung cancer with multiple pulmonary metastases. Additionally, multiple liver metastases were observed, with the largest measuring about 2.1x1.3cm. The patient was diagnosed with stage cT1bN3M1c IVB based on biopsy from the tumor in the right lung. Real-Time PCR indicated the presence of *EGFR* 19 E746_A750del(1) deletion mutation. As the first-line treatment, the patient received furmonertinib (80 mg once daily) for 27 months. A follow-up chest CT scan revealed an enlargement of the right lung nodule to approximately 2.6x2.1cm. Subsequently, the patient underwent 4 cycles of chemotherapy with pemetrexed combined with cisplatin. One month later, the chest CT scan showed the development of massive pleural effusion on the left side and enlarged liver lesions, measuring about 5.5x4.6cm. PCR genetic testing of tumor liver tissue revealed a mutation in *V600E* in exon 15 of the *BRAF* gene. The patient was then treated with dabrafenib (150 mg twice daily) and trametinib (2 mg once daily), and a follow-up CT scan after eight weeks showed improvement in the left pleural effusion and liver metastasis (**Figure 1**). So far, the patient responses well to targeted drugs and remains healthy with no signs of recurrence.

## Case 2

**Figure 2:**
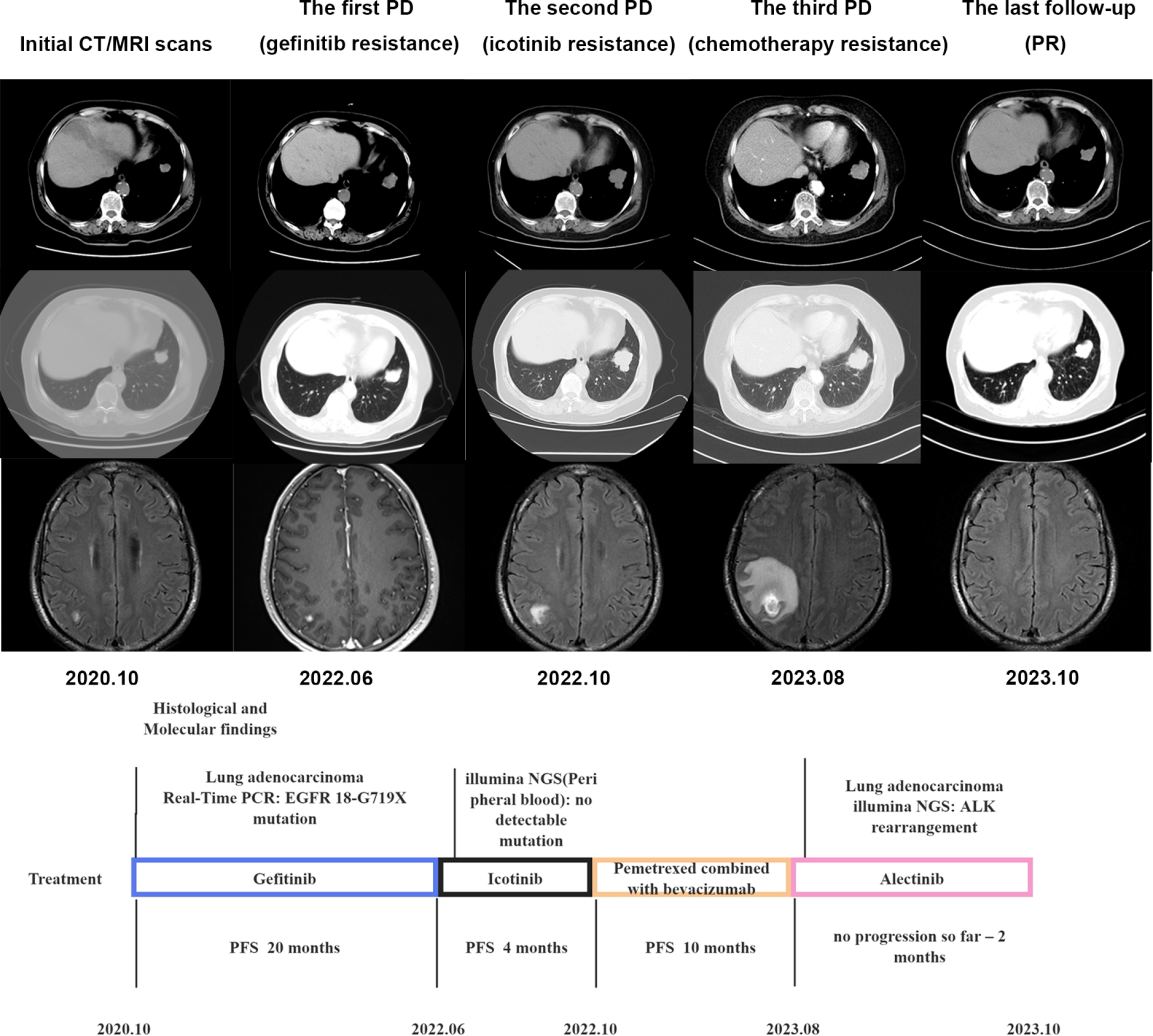
Timeline illustrating the changes in therapeutic regimen in correlation with molecular and radiological findings. PCR, digital polymerase chain reaction; PFS, progress free survival; NGS, next-generation sequencing; PD, progression of disease; PR, partial remission.

A 77-year-old female non-smoking patient presented with a cough. Imaging examination revealed the presence of nodules (1.7x1.5 cm in size) in the left lower lobe, along with multiple brain metastases. Genetic testing revealed a mutation in *EGFR*18-G719X. The patient was initially treated with gefitinib (250 mg once daily) with good tolerance and treatment for 20 months. NGS analysis of peripheral blood showed no mutations. Subsequently, the treatment was changed to icotinib, but a re-evaluation after 4 months demonstrated progression of the lung and brain metastases. Then the patient was treated with 6 cycles of Pemetrexed and Bevacizumab. The following MRI of the brain revealed enlarging lesions with extensive adjacent edema. Rebiopsy from progression lung tumor investigated by illumina NGS showed the presence of *ALK*-fusion, leading to the initiation of treatment with Alectinib. After eight weeks, reexamination showed a reduction in the size of lung lesions and disappearance of brain lesions (**Figure 2**). So far, the patient remains stable with no signs of recurrence.

## Discussion

We present two cases of advanced lung adenocarcinoma with two *EGFR* -mutations at initial diagnosis. In both cases progression on *EGFR*-TKIs was observed, leading to the identification of acquired resistance mechanisms in form of *ALK*(EML4 exon20) fusion and *BRAF* mutation. Interestingly, both patients achieved long-term survival with the use of *ALK* and *BRAF* inhibitors. These findings offer valuable insights for selecting therapeutic drugs in patients with acquired *ALK* fusion and *BRAF* mutation.

Mechanisms of *EGFR*-TKI resistance include *EGFR* mutations such as C797S, T790M, G796D, G724S, and L718Q, with T790M being the most common (50-60%). Additionally, resistance can arise from *MET* amplification (20%), *HER2* amplification (13%), activation of bypass pathways through mutation or amplification of tyrosine kinase receptor genes like *c-MET*, *FGFR*, and *HER2*, mutations in downstream signaling genes like *KRAS*, *BRAF*, *PIK3CA*, and *RAS/RAF/MEK/ERK*, and transformation into small cell lung cancer (10%) (6). *ALK* fusion is also involved in mediating *EGFR*-TKI resistance and may occur as intrinsic or acquired resistance mechanism (7). Primary fusion occurs in patients with dual alteration of *EGFR* mutation and *ALK* fusion genes, with a prevalence ranging from 0.9% to 6% in NSCLC (8). For patients with double alteration, the effectiveness of *EGFR*-TKI or *ALK*-TKI may depend on the mutation level of the involved gene (9, 10). Secondary fusion refers to *ALK* fusion may occur as a rare acquired resistance mechanism under treatment with *EGFR*-TKI *(* 11). However, cases of secondary *ALK* fusion following acquired resistance are rarely reported, and the optimal choice of targeted therapy remains inconclusive. In 2016, a case study reported the first patient with *EGFR* exon 19 deletion and *ALK* wild-type NSCLC. The study demonstrated the coexistence of primary *EGFR* mutation and acquired EML4-*ALK* gene fusion in plasma under *EGFR*-TKI treatment. The patient was treated with the *EGFR* inhibitor Osimertinib along with crizotinib, which resulted in sustained efficacy in the liver metastatic lesions (Extrahepatic lesions were stable) (12). In a study involving 3505 patients treated with *EGFR*-TKI, new *ALK*-fusions were detected in 7 patients. These fusion partners included EML4 (n=4), STRN (n=1), TGF (n=1), and PLEKHA7 (n=1). The patient with acquired STRN-*ALK* fusion did not experience the effect of Crizotinib. Additionally, in the patient with acquired PLEKHA7-*ALK* fusion, partial remission in six months was observed under the treatment with Alectinib and Osimertinib (13). Based on the hitherto published reports and the current case, combining of *EGFR*- and *ALK*-TKI may be an effective treatment in *EGFR*-mutated NSCLC with *ALK*-fusion acquired on the *EGFR*-TKI therapy. It is important to note that further research with a larger sample size is necessary to validate these results.

As one of the rare driver genes in NSCLC, *BRAF* mutations play a crucial role in the development and occurrence of tumors. Among these mutations, *BRAF V600* is the most commonly observed. With significant advancements in precision therapy, targeted therapy has emerged as a primary treatment approach for *BRAF* mutations. Dual-target combination therapy, in particular, has gained widespread recognition for its clinical value. This therapy involves the use of dabrafenib, a *BRAF* inhibitor, and trametinib, a *MAPK*-inhibitor. The combination of these two drugs allows for precise targeting of dual pathways and complete inhibition of the upstream and downstream pathways of *MAPK* (14). The results of the BRF113928 trial demonstrated an overall response rate (ORR) of 63.9% and a 5-year overall survival (OS) rate of 22% in first-line treatment with dabrafenib + Trametinib (D+T). Among the 36 subjects treated with D + T as after line, the ORR reached 68.4%, and the duration of remission (DOR) was 9.8 (95% CI 6.9-18.3) months. Furthermore, the trial confirmed the safety of this treatment regimen (15). The Chinese Lung Cancer Registration Clinical Study also provided evidence of the favorable antitumor activity and safety of dabrafenib + trametinib in Chinese NSCLC patients. Consequently, in 2023, dabrafenib and trametinib were recommended as grade I treatments in the Clinical Diagnosis and Treatment Guidelines of the Chinese Society of Clinical Oncology (CSCO).

*BRAF* mutation is also considered one of the mechanisms of *EGFR*-TKI resistance. However, the occurrence of BRAF mutation in *EGFR*-TKI resistance is rare, with an incidence of only about 1%. On the other hand, the rate of *BRAF* mutation after osimertinib resistance is higher, ranging from 3% to 10% (16). There was a study (17) showing that a combination treatment of dabrafenib, trametinib, and osimertinib resulted in clinical remission with an overall response rate (ORR) of 80% in five *EGFR*+ patients who had received osimertinib as a primary treatment. This suggests that the combination treatment may have a better therapeutic effect for patients with *BRAF* mutation after *EGFR* resistance. However, it is important to note that this study had a small sample size and there were significant differences in patient baseline data. Therefore, further studies with larger sample sizes are needed to validate these findings.

## Conclusion

While TKIs are widely used in NSCLC treatment, the occurrence of drug resistance is inevitable. The resistance mechanisms of 3rd generation *EGFR*-TKIs are complex and lack specific treatment. *ALK*-fusion and *BRAF*-mutations, although rare, cannot be ignored as they may emerge as mechanisms of *EGFR*-TKIs acquired resistance in a small number of NSCLC-patients. To develop more accurate personalized treatment plans, it is crucial to continuously explore potential drug resistance mechanisms and for finding therapeutic options under *EGFR*-TKIs resistance. This will further optimize the application of targeted therapy in NSCLC and provide precise guidance for individualized treatment of patients.

## Data Availability

The datasets presented in this article are not readily available because of ethical and privacy restrictions. Requests to access the datasets should be directed to the corresponding author.
